# Editorial for Practical Step-by-step SYNAPSE VINCENT Rendering of Three-dimensional Graphics in Horseshoe Kidney with Bilateral Varicoceles

**DOI:** 10.31662/jmaj.2024-0191

**Published:** 2024-09-06

**Authors:** Taichi Kin

**Affiliations:** 1 Department of Medical Information Engineering, Graduate School of Medicine, the University of Tokyo, Tokyo, Japan

**Keywords:** fusion 3D image, medical image, segmentation, SYNAPSE VINCENT

Medical imaging data, such as computed tomography (CT) and magnetic resonance imaging (MRI), are among the most critical information for diagnosis and treatment planning. With advancements in medical imaging technology, the types and volume of data in clinical practice have increased significantly, imposing a substantial burden on doctors in terms of interpreting images and planning treatments. Clinicians must integrate vast amounts of medical imaging information spatially and temporally in their minds when making diagnoses or planning treatment. This approach can result in significant individual differences in the accuracy and appropriateness of diagnoses and treatment and present challenges in terms of reproducibility and information sharing. In response to these challenges, extensive research has focused on integrating various medical imaging data types and visualizing them as fusion three-dimensional (3D) images. Clinical applications of fusion 3D images are also expanding ^(1)^. Particularly in surgical planning, fusion 3D images are reportedly more beneficial than conventional cross-sectional images for diagnosis, education, and training ^(2)^.

Creating fusion 3D images involves using a variety of image-processing techniques. This includes selecting and acquiring original medical images, performing registration to align coordinate systems across different image datasets, segmentation to extract target tissues, and rendering visualizations of the processed results ^(3)^. Understanding these detailed image-processing techniques is challenging for busy clinicians while also being engaged in daily clinical work. Many of these techniques differ significantly from traditional radiological diagnostic methods, making it difficult for doctors to acquire the necessary knowledge and skills for creating fusion 3D images. Moreover, the quality of fusion 3D images varies greatly based on the experience and proficiency of the operator. Therefore, Kojo et al.’s article ^(4)^ is particularly significant as it outlines a step-by-step method for creating fusion 3D images using medical-device software and demonstrates its clinical utility.

Kojo et al. ^(4)^ clarified the clinical objectives of creating fusion 3D images and underscored the importance of determining which anatomical structures should be visualized in 3D. Given the abundance of medical imaging data and numerous image processing techniques available, it is crucial to decide which data to use and how to visualize them in 3D. The article also provides practical details such as computer and software specifications, imaging conditions for original data, methods for inputting data into SYNAPSE VINCENT, and specific segmentation techniques. Unlike many reports on fusion 3D images ^(3)^, Kojo et al.’s work offers detailed insights into image processing.

Segmentation is considered to be the most crucial aspect influencing the quality of fusion 3D images. Achieving high-precision fusion 3D images requires proficiency in using various segmentation tools available in the software. While large structures such as the skull, major blood vessels, and tumors can often be extracted automatically or semi-automatically using methods such as thresholding or region growing, current segmentation technologies, including artificial intelligence (AI), do not yet achieve clinical perfection for fully automated extraction of microstructures. Thus, fusion 3D images are typically created by combining automatic, semiautomatic, and manual operations ^(5)^. Therefore, the segmentation process involves a flexible combination of methods, and results can vary significantly depending on the software and operator. The detailed segmentation methods described in Kojo et al.’s article ^(4)^ provide valuable guidance for doctors interested in learning to create fusion 3D images.

Limitations of Kojo et al.’s study ^(4)^ include the absence of evaluation of the clinical utility of fusion 3D images, lack of consideration for optimizing and standardizing image creation methods, and lack of assessment of the efficiency and accuracy of creating fusion 3D images.

While learning to create fusion 3D images may initially appear daunting for busy clinicians, they may find it less challenging after a few attempts. In addition, creating fusion 3D images enhances doctors’ ability to interpret medical images and reduces the risk of overlooking findings by requiring careful observation of original 2D cross-sectional images.

As medical imaging technology continues to advance, the volume of medical image data that doctors handle will inevitably increase. Fusion 3D images have the potential to be a valuable tool for addressing this challenge. As computer technology becomes more accessible, doctors are expected to be aware of its capabilities, limitations, utility, and future potential.

## Article Information

### Conflicts of Interest

None

### Author Contributions

All authors fulfill the ICMJE authorship criteria.

### Approval by Institutional Review Board (IRB)

This article does not require approval from ethics committee.

## References

[1] Kin T, Nakatomi H, Shojima M, et al. A new strategic neurosurgical planning for brainstem cavernous malformation using an interactive computer graphics with multimodal fusion images. J Neurosurg. 2012;117(1):78-88. 10.3171/2012.3.JNS111541 22577751

[2] Ruparelia J, Manjunath N, Nachiappan DS, et al. Virtual reality in preoperative planning of complex cranial surgery. World Neurosurg. 2023;180:e11-8. 37307986 10.1016/j.wneu.2023.06.014

[3] Kin T, Nakatomi H, Shono N, et al. Neurosurgical virtual reality simulation for brain tumor using high-definition computer graphics: a review of the literature. Neurol Med Chir (Tokyo). 2017;57(10):513-20. 28637947 10.2176/nmc.ra.2016-0320 PMC5638778

[4] Kojo K, Kim J, Saida T, et al. Practical step-by-step SYNAPSE VINCENT rendering of three-dimensional graphics in horseshoe kidney with bilateral varicoceles. JMA J. 2024;7(4):471-486. 10.31662/jmaj.2024-0058 PMC11543326 39513055

[5] Yoshino M, Kin T, Shojima M, et al. A high-resolution method with increased matrix size can characterize small arteries around a giant aneurysm in three dimensions. Br J Neurosurg. 2012;26(6):927-8. 22712458 10.3109/02688697.2012.692840

